# Immediate implant placement following tooth extraction with simultaneous lateral sinus augmentation: a retrospective clinical study after at least 1 year of loading

**DOI:** 10.1186/s40729-021-00377-0

**Published:** 2021-09-16

**Authors:** Bok-Joo Kim, Chul-Hoon Kim, Jung-Han Kim

**Affiliations:** Department of Oral and Maxillofacial Surgery, College of Medicine, Dong-A University, 26, Daesingongwon-ro, Seo-gu, Busan, 49201 South Korea

**Keywords:** Dental implant, Immediate placement, Sinus augmentation, Survival rate

## Abstract

**Background:**

Lateral sinus augmentation is necessary when the residual bone height is insufficient in the posterior maxilla. Immediate implant placement following tooth extraction with lateral sinus augmentation will shorten the number of operations and treatment time.

**Purpose:**

To evaluate radiologic and clinical results for at least 1 year after loading in patients who underwent tooth extraction, implant placement, and lateral sinus augmentation at the same time.

**Materials and methods:**

We retrospectively evaluated 35 implants placed in 25 patients. Preoperative and postoperative CBCT were compared and analyzed for residual bone height (RBH) and increased bone height (IBH), the initial torque value (ITV), and the implant stability quotient (ISQ). A comparative evaluation was performed between a 1-stage (non-submerged) group and a 2-stage (submerged) group. After loading for at least 1 year, clinical and radiological evaluations were performed to evaluate the survival rate.

**Results:**

One of the 35 implants failed in osseointegration, and the remaining 34 showed successful results. The failure-free survival rate at 1 year was 97.06% (95% CI, 91.38-100.0%). The RBH ranged from 3.1 to 9.6 mm (mean, 5.62 ± 1.68 mm), and the IBH ranged from 3 to 15.3 mm (mean, 8.87 ± 2.74 mm). Among the RBH, ITV, ISQ, treatment period, final bone height, and failure evaluation by stage of implant placement, only ISQ showed statistical significance between the groups (*p* < .001). A comparison of RBH, ITV, and ISQ, regardless of group, showed that each value tended to increase, but there were no statistically significant differences.

**Conclusions:**

Immediate implant placement following tooth extraction with simultaneous lateral sinus augmentation is considered reliable even though the procedures had been performed at the same time.

## Background

Insufficient residual bone height is a common problem encountered in rehabilitation of the posterior maxilla with implant-supported restoration. The bone available for implant placement may be limited by the presence of the maxillary sinus pneumatization together with loss of crestal alveolar bone. A surgical sinus augmentation procedure can be the solution to the problem of a lack of bone height. This procedure has proved to be highly efficacious and predictable [1–4].

The two main approaches to sinus augmentation are transcrestal and lateral opening. As regards the transcrestal approach, it is less invasive than the lateral opening approach and proceeds in a one-stage, but there are also some disadvantages associated with it. The amount of bone that can be gained is usually less than what can be obtained with the lateral sinus augmentation technique due to its limited vision and approach [5]. In addition, the residual bone should be sufficient [6–8]. As for the lateral approach, whereas it is more invasive than the crestal approach, it offers the significant advantage that vertical bone augmentation can be obtained as desired by directly elevating the maxillary sinus membrane [9].

With sinus augmentation, if primary stability is achieved in the residual bone, an implant can be placed simultaneously, even with the lateral opening approach [10]. Simultaneous placement of an implant has the advantage of reducing the surgical stage and treatment period and maintaining the space required to be filled with graft material [11, 12].

Implant placement in the immediate post-extraction phase is another approach that can reduce the treatment period and number of surgical procedures and minimize patient morbidity thereby [13, 14]. In fact, several trials have demonstrated successful clinical outcomes with high survival rates and stable crestal bone levels, similarly, to delayed implant placement [15–17].

Lateral sinus augmentation is necessary when the residual bone height in posterior maxilla is insufficient and immediate implant placement following extraction with lateral sinus augmentation will shorten the number of operations and treatment time. The purpose of the present study was to demonstrate at least 1 years’ worth of radiologic and clinical results of the loading of implant-supported restorations in patients who had undergone immediate implant placement in a fresh extraction socket with simultaneous lateral sinus augmentation.

## Methods

### Patients and pre-surgical evaluation

**Fig. 1:**
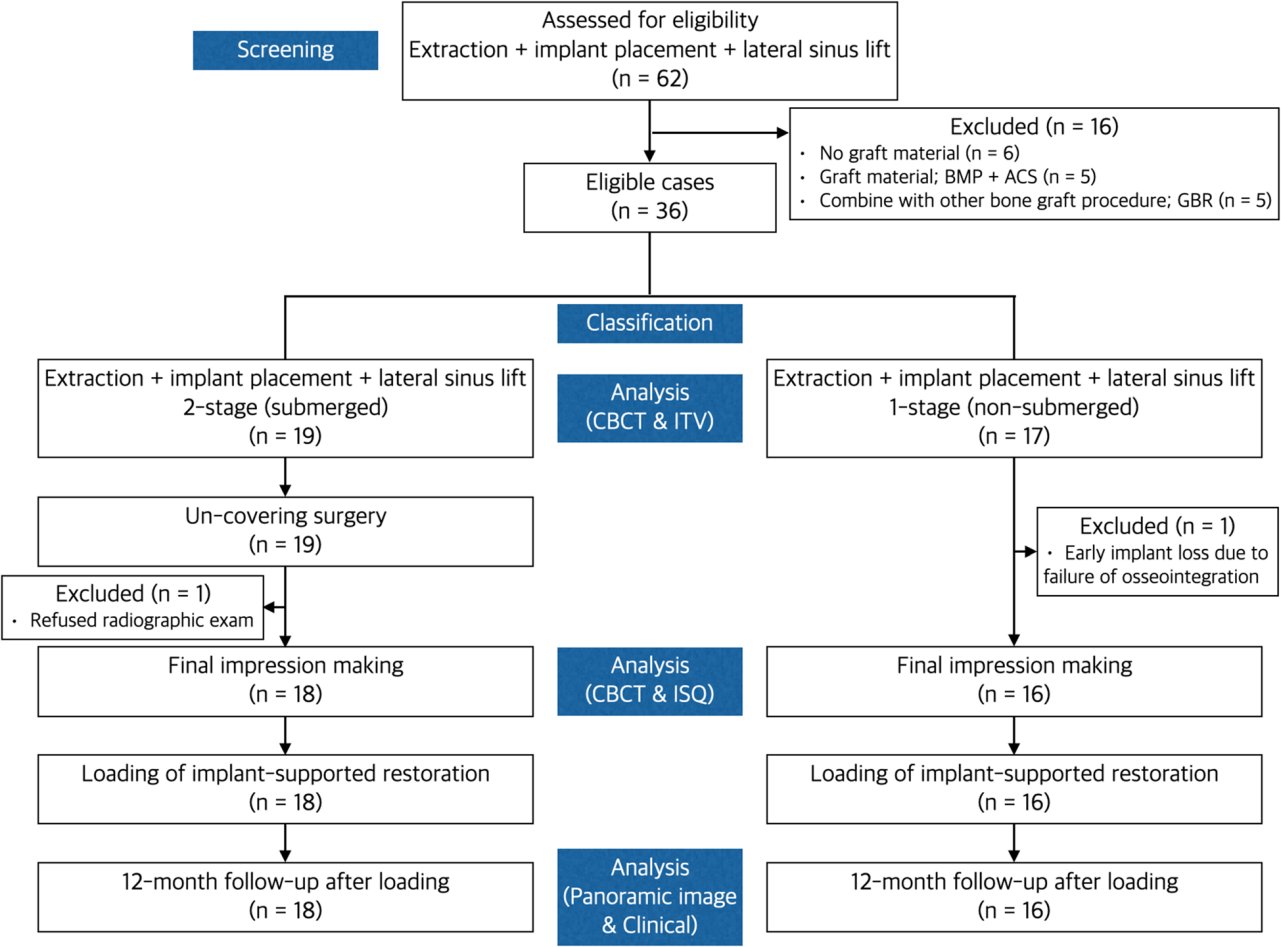
Flow diagram of patient selection, treatment, and evaluation

We retrospectively evaluated 25 patients (10 females and 15 males) aged 38 to 78 years (mean, 59.2 ± 11.6 years old) who had been treated at the Department of Oral and Maxillofacial Surgery at Dong-A University Hospital for tooth extraction and implant rehabilitation with lateral sinus floor augmentation between January 2015 and December 2017. A total of 35 dental implants were placed (Table 1 and Fig. 1). All of the participants signed an informed-consent form. A detailed explanation of each stage of the surgical procedure and the rationale for combining all of the surgical phases was provided. This study design complied with and was approved by the Ethics Committee of Dong-A University (IRB No. 2019-186). The guidelines of the Strengthening the Reporting of Observational Studies in Epidemiology were followed in this investigation. The surgical procedures were performed by two oral and maxillofacial surgeons (BJK and JHK) with more than 10 years of experience in dental implant surgery.

**Table 1:** Baseline characteristics of implants according to groups

		Group		
Variable	Overall (n = 35)	1-Stage (non-submerged) (***n*** = 17)	2-Stage (submerged) (***n*** = 18)	***p***
**Age (year)**				
Mean ± SD	59.63 ± 11.04	60.29 ± 12.32	59.00 ± 10.01	.631^2^
Range	35-78	38-78	35-71	
**Gender**				
Male	23 (65.7)	10 (58.8)	13 (72.2)	.404^3^
Female	12 (34.3)	7 (41.2)	5 (27.8)	
**Location of implant placement**				
First premolar	2 (5.7)	1 (5.9)	1 (5.6)	.146^4^
Second premolar	11 (31.4)	8 (47.1)	3 (16.7)	
First molar	8 (22.9)	4 (23.5)	4 (22.2)	
Second molar	14 (40.0)	4 (23.5)	10 (55.6)	
**Length of fixture (mm)**				
Mean ± SD	11.97 ± 0.12	11.94 ± 0.17	12.00 ± 0.00	.568^2^
Range	11.5-12	11.5-12	12-12	
11.5	2 (5.7)	2 (11.8)	0 (0.0)	.229^4^
12	33 (94.3)	15 (88.2)	18 (100.0)	
**Diameter of fixture (mm)**				
Mean ± SD	4.75 ± 0.39	4.78 ± 0.50	4.71 ± 0.24	.782^2^
Range	4.3-6.0	4.3-6.0	4.3-5.2	
4.3	11 (31.4)	7 (41.2)	4 (22.2)	.016^4^
4.8	17 (48.6)	4 (23.5)	13 (72.2)	
5.2	6 (17.1)	5 (29.4)	1 (5.6)	
6.0	1 (2.9)	1 (5.9)	0 (0.0)	
**Residual bone height (mm)**				
Mean ± SD	5.62 ± 1.68	6.06 ± 2.11	5.19 ± 1.02	.138^1^
Range	3.1-9.6	3.1-9.6	3.3-7.4	
**Increased bone height (mm)**				
Mean ± SD	8.87 ± 2.74	7.86 ± 3.04	9.77 ± 2.15	.040^1^
Range	3.00-15.30	3.00-14.90	6.20-15.30	

Values are either frequency with percentage in parentheses or mean ± standard deviation

^1^*P* values were derived from independent *t* test

^2^*P* values were derived from Mann-Whitney’s *U* test

^3^*P* values were derived from chi-square test

^4^*P* values were derived from Fisher’s exact test

Shapiro-Wilk’s test was employed for test of normality assumption

Preoperative panoramic radiographs and cone-beam computerized tomography (CBCT) were taken to evaluate the residual bone and sinus pathologies. None of the sinus pathologies were significant. The residual bone height of the edentulous site for implant placement ranged from 3.7 to 9.6 mm (mean, 5.62 ± 1.68 mm) (Table 1).

The teeth included in this study were cases of chronic periodontitis, deep caries, root rest, tooth fracture, and/or failure of endodontic treatment; none of them could any longer be preserved and required extraction and subsequent implant-supported restoration. The etiology of extraction included 15 cases of periodontal problems, 14 cases of tooth problems, and 6 cases of combined tooth/periodontal problems (Table 2).

**Table 2:** Etiology of tooth extraction

		Group		
Etiology	Overall (***n*** = 35)	1-Stage (non-submerged) (***n*** = 17)	2-Stage (submerged) (***n*** = 18)	***p***
**Periodontal problem**	15 (42.9)	6 (35.3)	9 (50.0)	.380^1^
**Tooth problem**	14 (40.0)	8 (47.1)	6 (33.3)	.407^1^
Caries	8 (57.1)	3 (37.5)	5 (83.3)	.138^2^
Root rest	5 (35.7)	5 (62.5)	0 (0.0)	.031^2^
Tooth fracture	1 (7.1)	0 (0.0)	1 (16.7)	.429^2^
**Combined periodontal and tooth problem**	6 (17.1)	3 (17.6)	3 (16.7)	1.000^2^

^1^*P* values were derived from chi-square test

^2^*P* values were derived from Fisher’s exact test

Shapiro-Wilk’s test was employed for test of normality assumption

### Inclusion and exclusion criteria

The following inclusion criteria were applied: (a) more than 18 years old; (b) immediate implant placement following tooth extraction with simultaneous lateral sinus floor augmentation within at least 1 year of loading; (c) lateral maxillary sinus augmentation using particulate bone graft material; (d) implant placement between 4 and 6 mm in diameter and between 10 and 12 mm in length; and (e) signing of informed-consent form and compliance with supportive maintenance therapy following surgical procedures. The following exclusion criteria were applied: (a) active infection or disease affecting bone and wound healing; (b) history of maxillary sinusitis or pathologies; (c) history of having undergone other bone augmentation techniques (e.g., guided bone regeneration); (d) history of lateral maxillary sinus augmentation using other bone graft material (e.g., no graft, bone morphogenic protein combined with absorbable collagen sponge); (e) current prescription medications possibly affecting bone metabolism, such as steroids, bisphosphonates, and medications for rheumatism (e.g., immunosuppressive agents); (f) history of head or neck radiation therapy; and (g) current pregnancy.

### Surgical procedure

The operation was carried out with the patient under local anesthesia (2% LidoHCl with 1:100,000 epinephrine). The perioral areas were aseptically prepared. A sulcular incision was made on the tooth to be extracted. A mesial and distal vertical releasing incision was made as needed. The flap was elevated carefully and extended labially to expose the bone. The mucosal flap was denuded subperiosteally to fully expose the sharp and thin alveolar bone and the lateral wall of the maxillary sinus. Extraction was carried out by careful application of elevators and forceps. If necessary, root separation was performed in multi-rooted tooth and roots were removed with a rotational and extrusionary motion to ensure minimal damage to the residual bony walls. After extraction, curettage and irrigation were performed on the extraction socket.

Extreme care was taken to radically elevate the sinus membrane from the lateral access window opened by using an electric-motor drill with appropriate water cooling. The floor and lateral, medial and posterior walls of the sinus membrane were meticulously detached and pushed upward to allow for the placement of implants into the bone chamber. The implant was positioned from the crestal bone and extended into the sinus, with primary stabilization provided by the residual bone, wall of the extraction socket and inter-radicular bone.

Two submerged implant systems (Zimmer, Zimmer Dental Inc., USA, and Dentis, Dentis Dental Inc., South Korea) were used. A mixture of demineralized freeze-dried bone allograft (OraGRAFT, LifeNet Health, Virginia Beach, VA, USA) and bovine bone xenograft (Cerabone, AAP Biomaterials GmbH, Berlin, Germany) was used as the bone graft material. Initial stability was measured with a hand toque wrench at the time of implant placement.

**Fig. 2:**
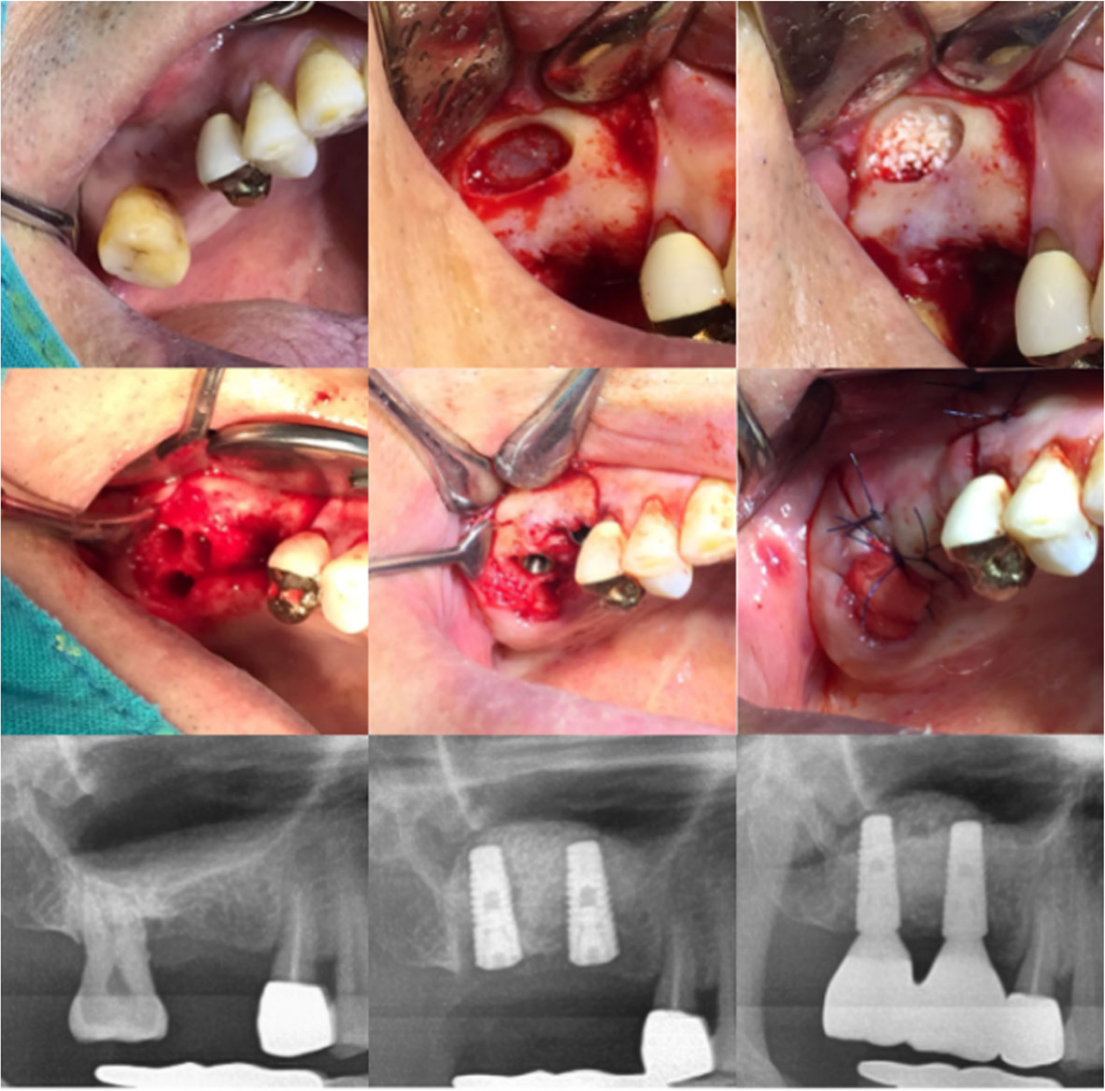
Clinical and radiographic aspects of immediate implant placement following tooth extraction with simultaneous lateral sinus lift: 2-stage (submerged)

**Fig. 3:**
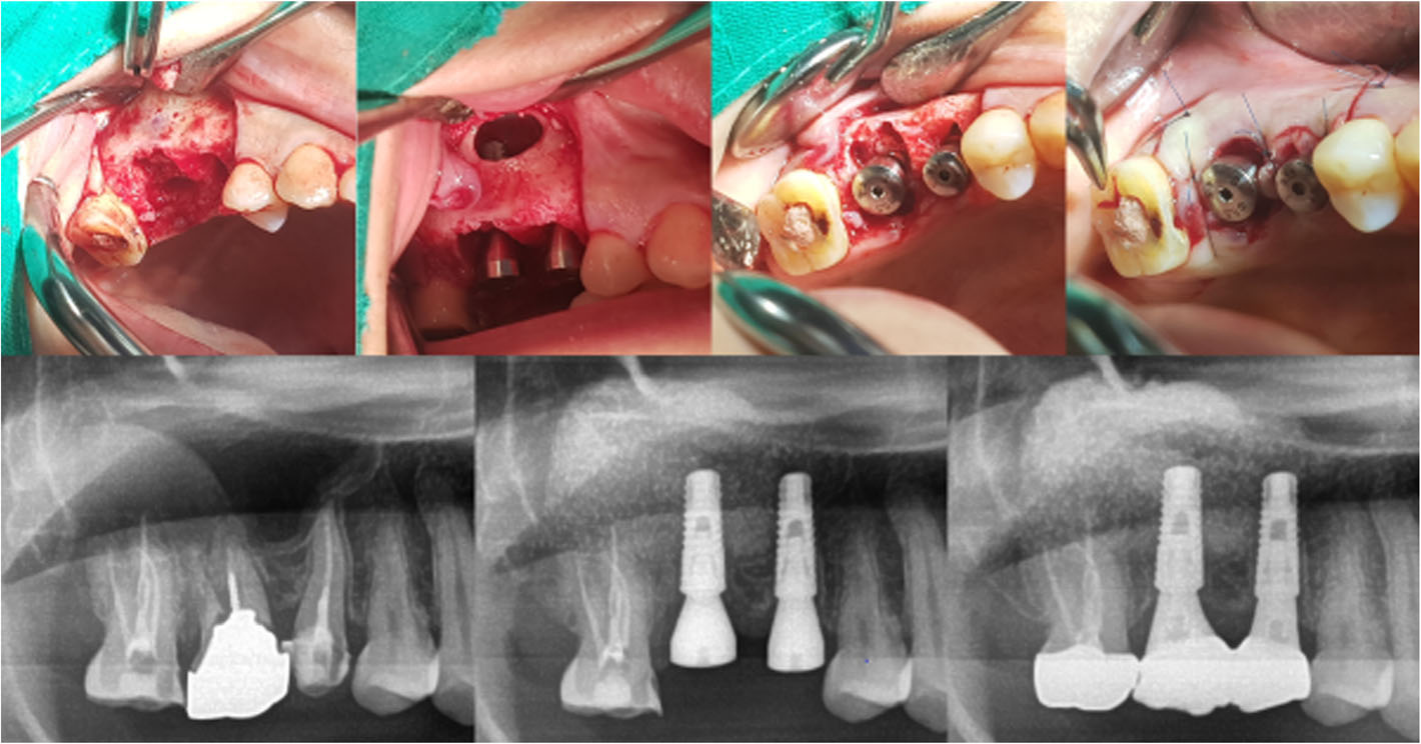
Clinical and radiographic aspects of immediate implant placement following tooth extraction with simultaneous lateral sinus lift: 1-stage (non-submerged)

The incision line was sutured by 5-0 nylon. After surgery, the patient received cephalosporins antibiotics of secondary generation, non-steroidal anti-inflammatory drugs and 0.1% chlorhexidine for 5 days. The suture was removed 7 days after surgery. The surgery proceeded via either a 1-stage (17 implants) or a 2-stage (18 implants) procedure. In the 2-stage procedure, a cover screw was connected to the implant, covered with a collagen sponge, and submerged (Fig. 2). In the 1-stage procedure, the healing abutment was connected to the implant and the surrounding soft tissue was approximated through sutures and non-submerged (Fig. 3).

The implants were subjected to 2-stage uncovering surgery after an average healing period of 168.3 days. The final impression of all of the implants (both 1-stage and 2-stage) was made after an average 180.5 days of implant placement. Implant Stability Quotient (ISQ) values were measured by Osstell Mentor (Integration Diagnostics AB, Göteborg, Sweden) at the final impression.

### Post-surgical evaluation

**Fig. 4:**
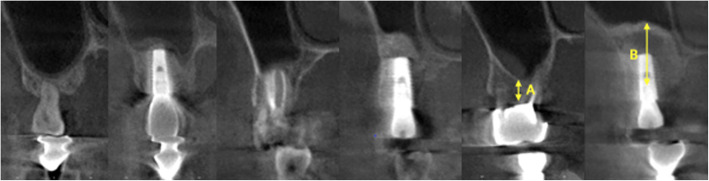
Cone-beam computerized tomography (CBCT) of patient shown in Figs. 2 and 3. **A** Residual bone height. **B** Increased bone height

At the time of the final impression, post-surgical CBCT was used to assess the bone formation, and the lifted bone height was measured as well. The preoperative and postoperative CBCT cross-sections of the implant position were measured, and the regenerated bone gained from the sinus elevation procedure between the primary cortical floor and the lifted sinus wall was measured (Fig. 4). The outcome of the dental implant was defined as “survival” when the prosthesis had been delivered and followed for at least 1 year without mobility, infection, pain, or more than 1.5 mm peri-implant bone loss in clinical exam and panoramic radiographs.

### Statistical analysis

The data are presented in either frequency (with percentage) for categorical variables or mean ± standard deviation (SD) for continuous variables. Differences in the study participants’ characteristics were compared across the subgroups by chi-square test or Fisher’s exact test for categorical variables and by independent test or Mann-Whitney’s *U* test for continuous variables, as appropriate. To determine the survival rate of the implant, the percentage and its failure-free survival (FFS) rate 95% confidence interval were calculated. Spearman’s correlation coefficients were used to assess the correlations among RBH, ITV, and ISQ. For the correlation coefficient, a score between −1 and 1 was reported (0 indicating no correlation, 1 signifying perfect positive correlation, and −1 signifying perfect negative correlation). All of the statistical analyses were carried out using SPSS 25.0, and *p* values less than 0.05 were considered statistically significant.

## Results

A total of 35 implants ranging from 4.3 to 6.0 mm in diameter (mean, 4.75 ± 0.39 mm) and 10 to 12 mm in length (mean, 11.97 ± 0.12 mm) were placed in the first premolar (2), second premolar (11), first molar (8), and second molar (14) areas. The height of the primary edentulous ridge below the sinus floor ranged from 3.1 to 9.6 mm (mean, 5.62 ± 1.68 mm). The increases in lifted sinus bone height ranged from 3 to 15.3 mm (mean, 8.87 ± 2.74 mm) (Table 1).

No patients developed maxillary sinusitis or infection. Sinus membrane perforation occurred in two patients and was repaired by application of a bioresorbable collagen membrane. One implant was removed 3 months after implant placement, due to a failure of osseointegration. After a 3-month healing period, it was replaced without additional bone grafting and reloaded 5 months later.

The remaining 34 implants healed well; no infection or implant mobility issue was detected on initiation of loading force from the prosthetic components. And after at least 1 year of follow-up after loading, the implants were maintained in a healthy condition and effectively supported the prosthesis. The failure-free survival rate at 1 year was 97.06% (95% CI, 91.38-100.0%) (Table 3).

**Table 3:** Failure-free survival rate of implant with 95% confidence interval

Survival rate of implant at 1 year	95% CI
97.06	91.38-100.0

The mean value of initial torque was 30.14 N/cm and ranged from 15 to 35 (15.1, 20.1, 25.8, 30.11, 35.14). The mean ISQ was 76.68 and ranged from 60 to 90 (mean, 76.68 ± 7.41). Among RBH, ITV, ISQ, treatment period, final bone height, and failure evaluation by stage of implant placement, only ISQ showed statistical significance between the groups (*p* < .001) (Table 4).

**Table 4:** Residual bone height (RBH), initial torque value (ITV), implant stability quotient (ISQ), treatment period, and failure by stage of implant placement

		Group		
Variable	Overall (***n*** = 35)	1-Stage (non-submerged) (***n*** = 17)	2-Stage (submerged) (***n*** = 18)	***p***
**RBH**				
Mean ± SD	5.62 ± 1.68	6.06 ± 2.11	5.19 ± 1.02	.138^1^
Range	3.1-9.6	3.1-9.6	3.3-7.4	
**ITV**				
Mean ± SD	30.14 ± 5.07	30.88 ± 5.66	29.44 ± 4.50	.222^2^
Range	15-35	15-35	20-35	
15	1 (2.9)	1 (5.9)	0 (0.0)	.342^3^
20	1 (2.9)	0 (0.0)	1 (5.6)	
25	8 (22.9)	3 (17.6)	5 (27.8)	
30	11 (31.4)	4 (23.5)	7 (38.9)	
> 35	14 (40.0)	9 (52.9)	5 (27.8)	
**ISQ**				
Mean ± SD	76.68 ± 7.41	81.94 ± 4.45	72.00 ± 6.32	<.001^1^
Range	60-90	75-90	60-85	
**Final impression (days)**				
Mean ± SD	180.47 ± 51.11	170.13 ± 53.70	189.67 ± 48.33	.201^2^
Range	86-346	86-302	131-346	
**Success or failure**				
Success	34 (97.1)	16 (94.1)	18 (100.0)	.486^3^
Failure	1 (2.9)	1 (5.9)	0 (0.0)	

Values are either frequency with percentage in parentheses or mean ± standard deviation

^1^*P* values were derived from independent *t* test

^2^*P* values were derived from Mann-Whitney’s *U* test

^3^*P* values were derived from Fisher’s exact test

Shapiro-Wilk’s test was employed for test of normality assumption

A comparison of RBH, ITV, and ISQ, regardless of group, showed that each value tended to increase, but there were no statistically significant differences (Table 5).

**Table 5:** Spearman’s correlation coefficients of residual bone height (RBH), initial torque value (ITV), and implant stability quotient (ISQ)

Variables	RBH	ITV	ISQ
**RBH**	1	.070 (.691)	.214 (.224)
**ITV**	.070 (.691)	1	.315 (.070)
**ISQ**	.214 (.224)	.315 (.070)	1

Values are Spearman’s rank correlation rho (*p* value)

There were no significant correlations among RBH, ITV, and ISQ (*p* > .05)

## Discussion

In cases of insufficient residual bone height due to crestal bone loss by periodontitis and maxillary sinus pneumatization, the traditional treatment is implant placement or delayed implant placement with simultaneous sinus augmentation after completion of extraction socket healing. Immediate implant placement after extraction has a similar success rate to that for cases of implant placement when socket healing is already complete [15–18]. Each of the above procedures has an excellent success rate. In the present study, with the goal of enhancing both treatment efficiency and patient convenience, we evaluated the results of immediate implant placement following tooth extraction with simultaneous lateral sinus augmentation.

Sinus augmentation can be achieved through either a transcrestal approach or a lateral opening approach. The transcrestal approach is advantageous in that the amount of flap elevation is small and the surgical damage is low, due to access through the implant osteotomy site [19]. However, this approach is a blind technique and has a disadvantage in that the amounts of lift and bone formation are limited. By contrast, the lateral approach is performed by directly elevating the maxillary sinus membrane, thus increasing the amounts of lift and bone formation. The results of this study, the increases in lifted sinus bone height ranged from 3 to 15.3 mm (mean, 8.87 ± 2.74 mm).

There are many studies on bone graft materials used in sinus augmentation [20–23]. In the present study, allograft was mixed with xenograft, and no problems were caused by this bone graft material. Sinus membrane perforation is the most common complications of sinus floor augmentation with an incidence of 8-24% [24]. When the sinus membrane elevation is performed immediately after tooth extraction, there may be difficulties in the procedure due to the irregularity of the floor of the maxillary sinus according to the shape of the root. On the other hand, when the delayed implant placement, the procedure could be easier, but there is a disadvantage in that the residual bone resorbed due to the pneumatization of the maxillary sinus. In this study, sinus membrane perforation occurred in two patients (2/25, 8%), but the perforation site was sealed with a bioresorbable collagen membrane and there was no drainage of the bone graft material into the maxillary sinus, and thus, neither maxillary sinusitis nor infection occurred [25–28]. Notably, then, it can be posited that immediate implant placement after extraction does not increase the incidence of sinus membrane perforation and maxillary sinusitis.

The advantage of immediate implant placement after extraction is that the implant can be placed at the same time as the extraction, reducing the number of operations and the total length of the treatment period, guide for implant placement and utilizing the healing mechanism of the extraction socket [16, 29, 30]. However, the disadvantage of this approach is the relative difficulty of obtaining primary stability due to the bone defect of the extraction socket [31]. The primary stability, rather, is obtained mainly from the residual bone. Additional primary stability can be achieved through the lateral wall of the extraction socket, in the case of a single-rooted tooth, or the lateral wall of the socket and the inter-radicular bone, in the case of a multi-rooted tooth. Especially in this case, additional primary stability can be obtained by using the cortical bone of the sinus floor through the lateral sinus lift. In the present study, the mean value of the initial torque was 30.1 N/cm, and primary stability was sufficiently obtained. The RBH and ITV differences were not statistically significant.

Another consideration in performing immediate implant placement after extraction is the treatment of the gap between the implant and the extraction socket. It is known that spontaneous healing is insufficient when the gap is 2 mm or more [32, 33]. Additionally, the thickness of buccal bone significantly influenced the amount of crestal bone resorption [34, 35]. In this study, bone grafting was carried out to fill the gap in 16 such cases.

After implant placement, we could distinguish between the 2-stage (submerged) and 1-stage (non-submerged) cases according to whether the cover screw or healing abutment was connected. In cases of immediate implant placement after extraction, it is difficult to close the soft tissue through the autogenous tissue, due to the soft-tissue defect of the extraction socket. In 2-stage cases, an advance flap via releasing incision or soft tissue graft techniques may be needed to address the above problem. However, the disadvantage is either the depth of the vestibule is shortened, or a second donor site is required. In this study, the fresh extraction socket has self-healing ability, a collagen sponge was used to help the initial wound healing and prevent the initial leakage of the bone graft material in the 2-stage cases, and no complications such as implant exposure occurred. In the 1-stage cases, the soft-tissue defect was reduced by connecting healing abutment, and the around soft tissue was approximated to the healing abutment through the suture, whereby the normal healing process was achieved. In the 1-stage cases, 1 of the 17 implants failed in osseointegration, but in the evaluation between the two groups, the ISQ was higher in 1-stage cases and there was a statistical significance (*p* < .001). This result is considered to be due to the progression in the 1-stage when the residual bone quantity is sufficient, and the residual bone quality is favorable. In our study, there were no definite criteria for the decision to perform 1-stage or 2-stage, as a result, the residual bone height and initial torque values between the two groups showed slightly higher in 1-stage, but were not statistically significant. A comparison of RBH, ITV, and ISQ, regardless of group, showed that each value tended to increase, but there were no statistically significant differences. The limitations are few cases in each group and the failure of only one of 35 implants; it was difficult to statistically analyze the survival rate according to groups and variables.

Currently, it is agreed that implants can be successfully placed at the time of extraction in infected sites as long as the infection is removed and primary stability is achieved [36–38]. The one case of failure in this study had a residual bone height of 3.8 mm, and the rationale for the extraction was the alveolar bone destruction due to chronic periodontitis. Primary stability was favorable at 25 N/cm and progressed to the 1-stage. The implant was in the normal healing process, but it was removed due to mobility and pain during a follow-up check 3 months after implant placement. Following a healing period of 3 months after implant removal, the implant was reinserted without additional bone grafting, and after 4 months of normal healing, a prosthodontic procedure was performed. The cause of the early failure of this case is unclear, but it may have been due to insufficiency of residual bone height and infection of the existing residual bone. However, there was no effect on the bone newly formed through lateral sinus augmentation.

After at least 1 year of loading, 1 of the 35 implants failed in osseointegration, and the remaining 34 showed successful results. The failure-free survival rate at 1 year was 97.06% (95% CI, 91.38-100.0%). The advantage of immediate implant placement after extraction with sinus lift is that all of the procedures are completed in one operation, with the result that the treatment period is short, and the patient’s discomfort is ameliorated. In this study, the time taken from implant placement to final impression was 180.5 days on average. This resulted in shorter edentulous periods and higher patient satisfaction.

The main limitation of our study is that the range of statistical survival rates is wide as 91.38-100.0% (95% CI) due to the insufficient number of study cases. In addition, the evaluation period was at least 1 year after loading, and long-term evaluation was not conducted. In the long-term maintenance of dental implant, vertical bone formation is important, but horizontal crestal bone change is also important, and this study did not evaluate it. But, within the limitations of this study showed that immediate implant placement in a fresh extraction socket with sinus lift was successful and that the incidence of complications, remarkably given the simultaneity of the procedures, did not increase. Further studies with a larger number of cases and longer follow-up periods will be needed.

## Conclusions

We evaluated the results of immediate implant placement following tooth extraction with simultaneous lateral sinus augmentation after at least 1 year of loading. Within the limitations of the study, immediate implant placement following tooth extraction with simultaneous lateral sinus augmentation is considered reliable even though the procedures had been performed at the same time.

## Data Availability

The datasets used and/or analyzed during the current study are available from the corresponding author on reasonable request.

## Abbreviations

RBH: Residual bone height
IBH: Increased bone height
ITV: Initial torque value
ISQ: Implant stability quotient
CBCT: Cone-beam computerized tomography
